# A value hierarchy for inclusive design of heart valve implants in regenerative medicine

**DOI:** 10.1080/17460751.2024.2357500

**Published:** 2024-06-10

**Authors:** Anne Johanna De Kanter, Manon Van Daal, Callum J Gunn, Annelien L Bredenoord, Nienke De Graeff, Karin R Jongsma

**Affiliations:** a Department of Bioethics & Health Humanities, Julius Center for Health Sciences & Primary Care, University Medical Center Utrecht, Utrecht University, Utrecht, 3508 GA, The Netherlands; b Erasmus School of Philosophy, Erasmus University Rotterdam, Rotterdam, 3062 PA, The Netherlands; c Department of Medical Ethics & Health Law, Leiden University Medical Center, Leiden University, Leiden, The Netherlands

**Keywords:** bias, bioethics, design requirements, diversity, heart valve implants, inclusion, *in situ* tissue engineering, justice, norms, values hierarchy

## Abstract

**Aim:** This paper investigates the conditions for inclusive design of regenerative medicine interventions from a bioethical perspective, taking regenerative valve implants as a showcase.

**Methods:** A value hierarchy is construed to translate the value of justice into norms and design requirements for inclusive design of regenerative valve implants.

**Results:** Three norms are proposed and translated into design requirements: regenerative valve implants should be designed to promote equal opportunity to good health for all potential users; equal respect for all potential users should be shown; and the implants should be designed to be accessible to everyone in need.

**Conclusion:** The norms and design requirements help to design regenerative valve implants that are appropriate, respectful and available for everyone in need.

## Plain Language Summary

Scientists in the field of regenerative medicine are developing a new type of heart valve implant. After implantation, the synthetic implant slowly breaks down and is replaced by a new living heart valve. These so-called regenerative implants promise a complete cure. However, they also raise ethical questions. For example, questions related to justice and inclusion. In this paper, we explore how regenerative implants can be designed to be inclusive, meaning suitable, respectful and available for everyone. We argue that the design of regenerative implants should be adapted to relevant differences between users. The implants should be affordable in the short and long term. The implants should be suitable for use worldwide. The implants should be designed by teams of diverse age, gender and ethnicity. Users should be engaged in the design. And the communication about the implants to researchers and users should be inclusive. Overall, this paper provides ethical guidance to researchers and clinicians developing regenerative implants.

## Abstract (plain language summary)

Health research and care should be inclusive to address inequities in health. Inclusion also deserves attention within the rapidly evolving field of regenerative medicine. However, what inclusion means and what inclusive design should entail in the context of regenerative medicine is not clear.

This paper investigates the conditions for inclusive design of regenerative medicine interventions, taking regenerative valve implants (i.e., *in situ* tissue-engineered heart valves) as a showcase.

The aim of inclusive design relates to the value of justice. Justice is concerned with recognition (claims about equal respect for marginalized groups and undervalued social identities) and redistribution (the equitable distribution of goods and resources).

A values hierarchy is a deliberative tool to translate a value into norms and design requirements in the context of technology design.

The value of justice can be translated into three norms: regenerative valve implants should be designed to promote equal opportunity to good health for all potential users; in the design process, equal respect for all potential users should be shown; and the implants should be designed to be accessible to everyone in need.

Based on these norms, the following design requirements can be formulated: regenerative valve implants should be designed to account for relevant user differences, be affordable and cost-effective and be suitable for global distribution, and the design process should involve diverse design teams, engage users and use inclusive communication.

Together, the norms and design requirements shape the conditions for inclusive design of regenerative valve implants.

This paper aids the design of implants that are appropriate, respectful and available for everyone in need. More generally, by exemplifying an ethically proactive approach to the design of regenerative medicine technology, it contributes to responsible development of regenerative medicine interventions.

## 1. Background

There is increasing acknowledgement that health research and care should be inclusive to address inequities in health. This plea is sustained by the growing evidence of health disparities between social groups, both locally and globally and the understanding that these disparities are related to social inequities (i.e., social determinants of health) and injustices [1,2].

Over the last decades, issues of quality and accessibility of healthcare have received much consideration, and recently there has been growing attention for the role of health technology. The design of health technologies and medical devices is one area of health research and care where social inequities and injustices are exemplified [3]. An approach to countering such inequities and injustices in the design of technologies is known as inclusive design. Inclusive design is broadly described as designing technologies and services that are usable by or appropriate for a broad range of individuals [4–6]. This broad range of individuals includes user groups that are sometimes ignored, particularly people from marginalized groups, e.g., those marginalized by disability, sex, gender, race, ethnicity or age. While inclusion of these groups matters throughout the entire process of technology development, implementation and utilization, this paper highlights the need for inclusive design at the early stages of technology development. In this phase, design plays a pivotal role in shaping decisions and practices, regarding for example the properties of the material, the intended purpose and use of the technology, and the user group(s) whom the technology is meant to serve. As such, inclusive design can contribute to ensuring justice in terms of who can benefit from an application, who is recognized as the beneficiary, as well as who can access it.

### 1.1. The need for inclusive design in regenerative medicine

Since the aim of regenerative medicine (RM) has been defined as to “*replace or regenerate human cells, tissues or organs, to restore or establish normal function*” [7], inclusion and inclusive design are deserving of special attention. Injustices from the past and present teach us that we have to be sensitive to how normal function or normality is defined. In medical research in general, the white, middle-aged male has long been taken as the dominant standard for bodily functioning [8–12]. This has led to severe historic and ongoing injustices, particularly harming people who deviate from this standard [13,14].

Although RM promises to overcome the barriers and biases of standardized medical research by offering personalized treatments, biases might still be present in the field of RM. For example, RM interventions are almost exclusively developed in the so-called ‘Global North’ (a term that notably oversimplifies the complex global landscape [15,16]) [17–19]. Currently, North America has the leading position in the global RM market [17], though research and development activities are gaining momentum in other regions, including Europe, Japan, South Korea and India [17,19]. Due to this focus on the development of RM interventions mainly in the Global North, there might be a selective focus on body types, diseases and resources in this region. This could negatively impact the suitability of RM interventions for the Global South, both in terms of effectiveness as well as opportunities for distribution. Inclusive design practices could help to make sure that RM interventions are suitable for people with diverse body types across the world and as such can serve everyone in need. Despite an increased awareness of diversity and inclusion in (bio)medical research and innovation in recent years, these issues have received little explicit attention in the context of RM. What inclusion means in the context of RM interventions is as yet unclear. No studies have analyzed the way that technology design in RM should best address issues of inclusion.

To fill this gap, this paper investigates the conditions for inclusive design of RM interventions, taking regenerative valve implants as a showcase. Regenerative valve implants – also referred to as *in situ* tissue-engineered valves [20,21] – are a promising application for the treatment of people with heart valve disease. These cell-free synthetic implants break down in the body and are replaced by living tissue. As such, they foster the growth of a living healthy heart valve [22,23]. While not yet clinically available, the implants promise to provide a life-long cure and to become an off-the-shelf available and cost-effective treatment. They are presented as alternatives for current valve replacement options with mechanical, biological or cryopreserved homograft valve implants [22,24,25]. Potentially, the implants could address an unmet clinical need for children and older adults in the Global North and younger adults in the Global South (Box 1).

Box 1. **Target populations of regenerative valve implants.**

There are several populations with heart valve disease for whom regenerative valve implants could potentially address an unmet clinical need. In the Global North, two main target groups can be discerned. One consists of children and young adults with congenital heart valve disease. Children currently require re-operations every few years to receive a correctly sized implant [24,26]. This group is much in need of a valve tissue that can grow with their body to provide a durable lifelong cure, which regenerative valve implants promise to provide. The other, much larger target group in the Global North are older adults with degenerative heart valve disease. Available options for valve replacement (biological valve implants) suffice for most of this group. However, adults with a relatively long life expectancy could benefit from a more durable implant, because this would prevent the need for reoperation associated with biological valve implants. Globally, a significant part of the population with heart valve disease lives in the Global South [27]. They suffer from heart valve disease caused by rheumatic fever and are on average much younger and have fewer comorbidities than the older adult population in the Global North [20,27]. Because many people in the Global South currently lack access to cardiovascular healthcare, this population is in need of affordable, accessible and effective treatment. It has been suggested that Regenerative Medicine implants could meet this need [22].

## 2. Methods

### 2.1. Approach: a value hierarchy

In this paper, we investigate the conditions for inclusive design of regenerative valve implants from a bioethical perspective. We understand design to refer to the technical aspects of the technology as well as to the organizational and procedural aspects of the design process.

To investigate the conditions for inclusive design of regenerative valve implants, we construe a value hierarchy. A value hierarchy or values hierarchy is a deliberative tool to translate one or more values into norms and design requirements in the context of technology design [28]. It can be used to address which values are expressed in a design and to explicate how the design is shaped by these values. The value hierarchy consists of three layers, i.e. values, norms and design requirements, which each can have several sublayers [28]. We construe a value hierarchy for inclusive design of regenerative valve implants, starting with the value of justice. In part I, we explore the relation between the aims of inclusive design and the value of justice, and we conceptualize justice. In part II, we translate the value of justice into norms relevant for the design of regenerative valve implants. In part III, we translate each norm into design requirements. Together, the norms and design requirements shape the conditions for inclusive design of regenerative valve implants.

Overall, by laying down the conditions for inclusive design of regenerative valve implants, we hope to aid the development of regenerative valve implants that are appropriate, respectful and available for everyone in need. More generally, by exemplifying an ethically proactive approach to the design of RM technology, we hope to contribute to responsible development of RM interventions.

## 3. Results

### 3.1. Justice as a value for inclusive design

The aims of inclusive design are closely related to the value of justice [4,29]. A design that is inappropriate for groups of potential users will be exclusive, and it has been argued that if this exclusion deprives these groups of capabilities, they have good reason to desire, this may be considered inequitable and therefore unjust [4].

**Figure 1.**
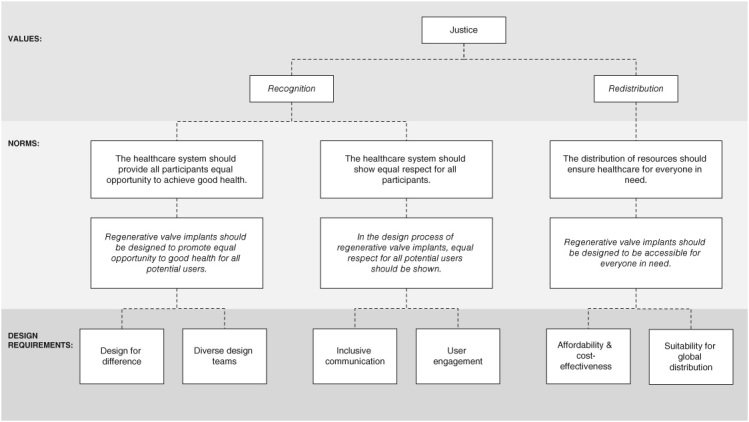
A graphical presentation of the value hierarchy for inclusive design of regenerative valve implants.

Justice has been conceptualized in many different ways [30,31]. We draw here on the conceptualization of justice as proposed by feminist philosopher Nancy Fraser, who has argued that justice is concerned with both recognition and redistribution [32]. These two dimensions of justice are integral to shaping inclusive design practices. Recognition refers to claims about equal respect for marginalized groups and undervalued social identities [32]. Justice as recognition should inform inclusive design practices because equal respect and equal opportunity for all users are prerequisites for inclusive practices and form the basis for creating a design that fits the needs and wants of all users of a technology. Redistribution refers to the equitable distribution of goods and resources [32]. Inclusive design practices should also be informed by justice as redistribution because equal access to a technology is a prerequisite for inclusive practices, and the possibilities for access are in part determined by elements in the design. Accordingly, we start our value hierarchy from the value of justice, split into two sub-values, recognition and redistribution (Figure 1). See Box 2 for further detailing of Fraser's conceptualization of justice.

Box 2. **Fraser's conceptualization of justice.**

Central to Fraser's approach to justice is the norm of *parity of participation* [32]. Parity of participation prescribes that individuals in a society must be able to interact with each other as peers [32]. There are two preconditions for parity of participation. First, the *intersubjective precondition* prescribes that equal respect for all individuals and equal opportunity to achieve self-esteem should be institutionalized [32]. This precondition is closely connected to *justice as recognition*, which means that individuals hold the status of full partners in social interaction [33]. Misrecognition or status subordination occurs when social institutions lay down cultural norms that frame some as less than full members of society [33]. Examples of social institutions include governments, universities, hospitals, business corporations and legal systems [34]. Second, the *objective precondition* for parity of participation prescribes that resources must be distributed to ensure ‘individuals’ independence and voice’ [32]. This precondition is closely connected to *justice as redistribution*, which refers to equitable allocation of resources.

### 3.2. Specifying justice into norms

We translate the value of justice into norms, formulating two sublayers of norms. The first sublayer specifies three norms relevant for a healthcare context (Figure 1): *the healthcare system should provide all participants equal opportunity to achieve good health, and show them all equal respect and the distribution of resources should ensure healthcare for everyone in need*.

The second sublayer specifies three additional norms for inclusive design of regenerative valve implants (Figure 1): *regenerative valve implants should be designed to promote equal opportunity to good health for all potential users; in the design process of regenerative valve implants, equal respect for all potential users should be shown; regenerative valve implants should be designed to be accessible for everyone in need*. See Box 3 for further details on how the specification from the value of justice into norms took place.

Box 3. **Specifying the value of justice into norms.**

To bridge the gap from assessing justice to prescribing actionable norms, we formulate two sublayers of norms and draw inspiration from the Fraser's notion of *parity of participation* (Box 2).

The first sublayer specifies norms relevant for a healthcare context (Figure 1). The intersubjective precondition for parity of participation is closely related to the concept of *justice as recognition*, emphasizing the importance of providing all individuals with equal opportunities to achieve self-esteem and institutionalizing equal respect for them all [32]. Healthcare is an essential institution and offers a pathway to enhancing self-esteem by caring for one's health and well-being. We translated this into two norms: *the healthcare system should provide all participants equal opportunity to achieve good health, and show them all equal respect*. The objective precondition for parity of participation is closely related to justice as redistribution, emphasizing that resources should be distributed in a way that allows all individuals to be independent and to be heard [32]. Healthcare is one important way to allow this. We translated this into the following norm: *the distribution of resources should ensure healthcare for everyone in need*.

Second, we specified these three norms, which are still quite broad, into more specific norms for inclusive design of regenerative valve implants (Figure 1). Regenerative valve implants are a particular kind of healthcare. The specific norms then are: *regenerative valve implants should be designed to promote equal opportunity to good health for all potential users; in the design process of regenerative valve implants, equal respect for all potential users should be shown; regenerative valve implants should be designed to be accessible for everyone in need*.

#### 3.2.1. Recognition, difference & bias

The first two norms, related to *justice as recognition*, raise the question of how to deal with biases. Neglecting or invalidating differences between people can result in (normative) bias within a healthcare context. This can be exemplified by Ruiz & Verbrugge's definition of gender bias: gender bias systematically affects healthcare by assuming similarities in health determinants between women and men when differences exist, and by presuming differences where there are in fact similarities [35]. Therefore, bias, in general, poses a dual challenge in the recognition of differences [36]. On the one hand, bias can occur when differences between individuals are not recognized and acknowledged despite their relevance to the situation or context at hand. On the other hand, bias can occur when differences between individuals are given undue significance or influence while they are not relevant for the given situation or context. We will return to this distinction in the next section.

### 3.3. Specifying norms into design requirements

We specify each norm into design requirements for regenerative valve implants (Figure 1). Below, we substantiate each design requirement and provide recommendations regarding what is needed – if anything – to meet each requirement. Together, the norms and design requirements shape the conditions for inclusive design of regenerative valve implants.

#### 3.3.1. Norm: Regenerative valve implants should be designed to promote equal opportunity to good health for all potential users

##### 3.3.1.1. Design requirement: design for difference

To promote equal opportunity to achieve good health, regenerative valve implants should fit the needs of every person. In other words, the implants should be designed to be appropriate for everyone in need. With ‘appropriate’ we refer not only to the treatment being (medically) effective for a user or user group, but also it being suitable in broader terms, for example whether the properties match the user's or user group's social context or behavioral characteristics [cf. 4]. This design requirement necessitates that relevant differences between users are taken into account in the implant design.

Relevant user differences may include biological differences and differences in social identities. Biological differences such as age, sex and comorbidities could be relevant for the implant design for different reasons. For example, the size of the heart is co-dependent on age and sex, and the size of the implant might need to be adapted accordingly. Moreover, biological differences might affect the regeneration response, and could therefore co-determine the effectiveness of the treatment. Thus far research on these effects is limited, yet preliminary data suggests that age, sex and comorbidities might affect the regeneration response [23,37]. Differences in social identities, such as age, gender and race/ethnicity and related differences in social context, lifestyle and behavioral factors, might also affect the success of the treatment and its overall impact on the user's life. For example, alcohol and drug abuse, which are lifestyle factors that are linked to gender [38], have been found to affect the regenerative capacity of bone tissue [39]. A similar effect for valve regeneration is to be considered. Currently, research into the significance of social differences for regenerative valve implants is scarce.

It has been suggested that, if necessary, the design of regenerative valve implants could be adapted to match different user groups [23,37]. This approach is called *stratification* or precision engineering. To determine if stratification is necessary, more research should be done into whether and how biological and social differences affect the appropriateness of regenerative valve implants for different user groups. Such research is especially important given that the potential user population of valve implants in the Global South differs significantly from those in the Global North: the former is on average younger, has fewer comorbidities and valve disfunction is generally caused by a different type of disease [27]. Considering if and how these differences impact the appropriateness of regenerative valve implants, and, when necessary, adapting the implant design, accordingly, is essential. This approach ensures that this treatment is both responsive and inclusive of the needs of all potential users.

In designing for difference, three important considerations should be taken into account. First, the distinction between social identities and biological differences is far from absolute and clear, and they overlap in many ways. For example, age is both a biological characteristic, as well as a social identity, considering that aging can refer to physical changes in one's body as well as to changes in the person's roles, identity and relationships. With regards to sex and gender, sex is usually taken to refer to biological characteristics, while gender is taken to refer to sociocultural characteristics. However, sex and gender influence each other, and sex is a social construct in itself [40]. With regards to race, in a biomedical context, race has often been mispresented as a biological characteristic, even though it is now widely accepted that race is a social construct with nonetheless very real consequences for people from many racial groups [41, p.200]. In short, race should be understood as a social identity and characteristics such as age and sex/gender can be both social and biological, and the significance of these aspects should be considered in the design of regenerative valve implants.

Second, it is important to consider when differences are significant and when they are not. As we argued above, bias might occur both when significant differences are not recognized, as well as when insignificant differences are given undue attention. It might be helpful to go beyond usual broad categories like race/ethnicity, sex/gender and age and look into more specific differences between people. In line with what we argued elsewhere, we propose that categories of difference should be defined by specific, causally significant and morally justifiable attributes (such as skin color, hormone levels, behaviors or lifestyles) rather than using broader categories (such as race or sex) as proxies. Previously, we argued that differences should be defined by ‘morally significant’ attributes [36]. In response to a thoughtful (published) commentary [42], we here adapt our formulation to the more accurate descriptions ‘causally significant’ and ‘morally justifiable’. Specific attributes can be causally significant if there is a clear causal relation with the device's functioning and if this causality has been scrutinized for (socio-historically rooted) biases [36].

Third, when accounting for differences between people, it is important to recognize that individuals within these categories of differences are not homogenous. Every person has several biological characteristics and social identities at the same time and therefore might have specific needs. This underscores the importance of adopting an intersectional approach. Intersectionality acknowledges how the intersection of multiple social identities impacts various aspects of human lives, including opportunities, inequities and inequalities. Accounting for intersectionality in regenerative valve implant design is important to ensure that the implants are appropriate for everyone within a specific category, whether it be women or men, young or old. For instance, the implants should benefit all women in need, not exclusively those in the Global North or those with a particular ethnicity. Therefore, research into human differences should also explore which and how different biological differences and social identities intersect. Moreover, if the design of regenerative valve implants moves toward a stratified approach, these intersections should be taken into account when user groups are defined.

##### 3.3.1.2. Design requirement: diverse design teams

To counter bias and to promote equal opportunity for all users, in the development of regenerative valve implants, design teams with engineers, clinicians and other developers should be diverse, for example in terms of gender, age, ethnicity, nationality, work experience and scientific discipline. Heterogeneous design teams are more likely to develop an inclusive design than homogeneous teams. For example, it is known that gender diversity enhances collective problem-solving capabilities within teams, stimulates effective use of the expertise of each team member, and leads to broader inquiries that address a wider range of topics and questions [43]. The impact of a lack of diverse design teams can be illustrated by Apple's Health app, where a male dominated team designed a health app that initially lacked features specific to women's health such as tracking the menstrual cycle [44,45]. This illustrates how (the interests of) certain populations can be forgotten when the engineering team is too homogenous.

Specific figures regarding gender diversity or ethnicity in RM development teams are not available. However, the numbers of women and individuals from marginalized communities working in both the broader STEM sciences and cardiovascular medicine indicate that certain groups are underrepresented, and this may extend to RM as well [46–48]. Incorporating a range of diverse viewpoints within the design team of regenerative valve implants is likely to positively impact inclusion in all aspects of research and development.

#### 3.3.2. Norm: In the design process of regenerative valve implants, equal respect for all potential users should be shown

##### 3.3.2.1. Design requirement: inclusive communication

Inclusive communication through language and visuals are two important ways to show equal respect to all users. Inclusive language refers to the words, phrases and metaphors that are used that should *“avoid expressions that might be considered to exclude particular groups of people”* [49]. Inclusive communication is crucial because it acknowledges the unique identities and backgrounds of all users, which reinforces that they are valued and worthy of respect. It also ensures that research descriptions and findings accurately reflect the characteristics within the population or groups being studied. Moreover, it helps developers avoid reinforcing stereotypes or perpetuating bias, whether related to gender, race, age, disability or other characteristics.

Therefore, in the design process of regenerative valve implants, developers and clinicians should pay attention to inclusion in the representation of biological differences and social identities of potential users in their written, verbal and visual communication. Language and visual communication should be sensitive to causally significant differences in gender, sexual identities, disability and ethnicity, among others [50], so that these representations accurately reflect a diverse range of potential users.

Examples of inclusive language relevant to the context of regenerative valve implants include referring to people with a disease or disability as *persons* (with disease or disability x) rather than patients, which is still the predominantly used term in RM literature; using gender-neutral terms when gender is irrelevant, for example to avoid terms like Mr. or Ms. unless the individual prefers them. Inclusive language also extends to the critical examination and re-evaluation of the concepts and theories that researchers use [51]. For instance, adhering to biased concepts and theories, such as associating heart disease primarily with older individuals (which was common in the past), may lead to overlooking the prevalence of heart diseases across all age groups. This can result in underdiagnosis and inadequate treatment for younger adults and even children.

**Figure 2.**
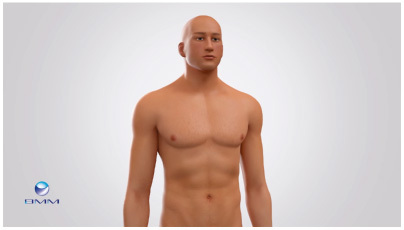
A still from an explanatory video on a regenerative heart valve replacement. The still depicts a person with heart valve disease, as is explained in the voice over. Reproduced with permission from [52].

In relation to inclusive visuals, an example from the RM field of what can go wrong can be found in an explanatory animation of heart valve implants, published in 2014 by a Dutch biomaterials consortium [52]. The body of a person living with heart valve disease is represented as that of a strong, white, young adult, able-bodied man (Figure 2). While one person can never be representative of a whole population, depicting the user with tenets of a historically dominant and privileged group perpetuates bias in favour of this group. While this is not necessarily representative of other communication about regenerative valve implants, as an example it emphasizes the need to pay attention to inclusive and representative visualizations in communication about regenerative valve implants.

Several strategies may facilitate inclusive communication. Existing inclusive language and visual guidelines can serve as a useful starting point [e.g., 50]. Partnerships may be established with healthcare advocacy groups to facilitate development of sensitive and accessible communication materials that resonate with potential users. Through these collaborations, communications strategies can be co-created to address the unique concerns and challenges encountered by diverse healthcare advocacy communities and to avoid perpetuating stereotypes. Moreover, developers and clinicians may be trained to develop skills to use inclusive language and create visuals that respect the diversity of individuals and avoid perpetuating stereotypes. Such training sessions should also underscore the significance of creating communication materials that are accessible to people with disabilities, which may include alternative formats, clear fonts and descriptive images.

Inclusive language and visuals should be integrated into the design process of regenerative valve implants from the very outset. This starts with the initial concept development and should continue throughout all phases of research and development, including pre-clinical experimental research, animal testing, early phase human trials and the implementation phase. Inclusive language and visuals should be used both in communication among developers and clinicians themselves, such as in grant proposals, consortium meetings and academic publications, as well as in communication to the users and broader publics, such as in information videos and folders and on websites. This communication should be inclusive to the different stakeholders for whom the regenerative valve implants are developed, such as potential users, i.e. broader publics (who are potential future users).

##### 3.3.2.2. Design requirement: user engagement

Showing equal respect for all potential users also entails that users' voices should be heard, and engagement of users is one important way of accomplishing this. User engagement can be established through direct collaboration with healthcare advocacy groups. An example in the Dutch context is the Hartstichting, which is a healthcare advocacy group for people with cardiovascular diseases. Healthcare advocacy groups can connect engineers and clinicians with potential users of regenerative valve implants to ensure that research includes relevant user perspectives. Potential users can provide input on for example unmet needs, what is important to them in terms of potential risks and benefits and whether they understand the information material related to human trials. These user perspectives can be used to identify and anticipate expectations, possible issues and wishes relating to regenerative valve implants and their impacts on the daily lives of potential users and their caregivers. In this way, user values and experiences can influence the design of the implants. By gathering a variety of perspectives and experiences, user engagement might also help to counteract biases in the design process of and communication about the implants. Users can be engaged during several stages of the development process, such as when setting research objectives, gathering and analyzing data and implementing results [53]. Ideally, the user sample involved in engagement activities should be representative of the potential user population and should be heterogeneous enough to capture the various intersecting positions of relevance to regenerative valve implants. Knowing which intersections are relevant requires a better characterization of the potential users of the implants. Engagement activities to inform the design process can then be set up that reach out to groups of users based on relevant characteristics. If selecting a representative user sample turns difficult to achieve in practice, paying attention to potentially relevant differences in user groups will at least elicit a more inclusive set of user perspectives relevant to the research and design process.

#### 3.3.3. Norm: Regenerative valve implants should be designed to be accessible for everyone in need

##### 3.3.3.1. Design requirement: affordability & cost–effectiveness

Affordability and cost–effectiveness of the implant treatment could become a major barrier for access to regenerative valve implants, particularly for the population in the Global South. To promote access to regenerative valve implants for everyone in need, the implants should therefore be designed to be affordable and cost effective.

While developers present regenerative valve implants as a relatively affordable treatment (at least in comparison with other RM treatments) [20,22], no cost assessment of the implant pricing and upfront implementation costs has yet been carried out [24]. Even if the implant pricing is similar to mechanical and biological implants (as assumed in some studies [21,54]), the potentially high upfront costs remain a concern in economically disadvantaged areas. For instance, the incorporation of regenerative valve implants into routine clinical contexts requires substantial financial investment, and medical staff needs specialized training to handle complex regenerative procedures. Affordability could be promoted by setting up investment funds to help hospitals in certain settings to be able to make necessary upfront investments and allow them to pay back these investments over time.

In addition to being affordable, the implant should also be cost effective. In health technology assessment, cost–effectiveness of a new treatment is determined based on a comparison of the costs and treatment outcomes with those of an existing treatment. Interventions are considered cost-effective if the long-term costs are lower, or if the improvement in treatment outcomes is sufficient relative to the additional costs. Assessing cost–effectiveness early on can be facilitated by economic evaluation as performed in early health technology assessment. It is beyond the scope of this paper to elaborate on the methods for assessing the cost–effectiveness in detail (which others have already done in more depths [55–58]). However, it is worth pointing out that assessing cost–effectiveness of regenerative valve implants might be challenging given their potentially high initial costs, uncertainty regarding the long term impacts (benefits and harms) and a lack of absolute comparator interventions [59].

Studies have indicated that regenerative valve implants can become a cost-effective treatment for children [54]. Even if the price of the implants turns out higher than currently available implants, the treatment could be cost-effective for children on the long-term provided that regenerative valve implants are more durable and are able to grow with the child's body [54]. This would reduce the need for re-operation and improve this group's quality of life. Unfortunately, growth potential of the implants has currently not yet been clinically demonstrated [25]. For older adults, particularly for the group below 80 years, treatment with regenerative valve implants can also become cost-effective, provided that regenerative implants are similarly priced as currently available implants and have improved durability and infection resistance [21]. This would also improve their quality of life.

If the implants are cost effective, this would improve accessibility in areas where universal health coverage is available, given that coverage is usually based on certain cost–effectiveness thresholds. By cutting the overall treatment costs, cost–effectiveness could also promote access for people in areas where health coverage is limited. However, it is worth noting that while regenerative implants may prove cost-effective in the long term, its upfront implementation costs may still render them unaffordable for healthcare systems and people with no or limited health coverage. Therefore, it is important that while assessing the affordability and cost–effectiveness, one should consider the local context and healthcare system characteristics. Factors such as reimbursement policies, healthcare infrastructure and potential user demographics may influence the affordability and cost–effectiveness of regenerative valve implants in different settings. This could mean that affordability and cost–effectiveness of regenerative valve implants may vary between, for instance, the Global South and Global North.

##### 3.3.3.2. Design requirement: suitability for global distribution

To ensure access to regenerative valve implants for everyone in need across the world, the implants should be suitable for global distribution. Given that the implants are currently mainly developed for target groups (children and older people) in the Global North (see introduction), particular attention should be paid to the needs of populations in the Global South. In these countries, the majority of the population has limited access to cardiac surgery in general [60] and to valve implants in particular [27]. While it has been suggested that the implants could become available worldwide [22], to date, there have been no substantial investigations to determine the requirements to distribute regenerative valve implants in countries in the Global South. Research should be done to map which requirements should be met to live up to this promise.

As a preliminary indication, in addition to affordability and cost–effectiveness, other design requirements to promote access for populations in the Global South, particularly those in rural areas, likely include that the implants should be designed to be easily storable and transportable and should be designed to be implantable by local surgeons in non-specialized hospitals. The first requirement is already met in the current design of regenerative valve implants, which are presented as ‘off-the-shelf available’ [24,25], meaning that (in contrast to for example stem cell-based RM treatments) they can be bulk-produced rather than custom-made and do not need highly specialized storage conditions. The second requirement is important because if regenerative valve implants would only be implantable in specialized centers, availability could be confined to large teaching hospitals or specific regions within a country. This would pose a significant barrier for people who are unable to travel from rural areas, such as older individuals or those who face socioeconomic challenges [61,62]. The implants have the potential to make a substantial impact on cardiovascular healthcare outcomes in the Global South, the field of RM should therefore anticipate the necessary actions needed to ensure health systems have the capacity to promote widespread availability of regenerative valve implants and deliver these to populations in need.

## 4. Discussion

In this paper, we have investigated the conditions to make the design of regenerative valve implants inclusive and aligned with the value of justice. To this end, we have proposed a value hierarchy based on a conceptualization of *justice as recognition* and redistribution, specified into three norms, each specified further into two design requirements (Figure 1). Together, these norms and design requirements establish the conditions for inclusive design of regenerative valve implants.

Our paper shows that values, in this case the value of justice, can be translated into conditions for value-informed practices in biomedicine. We focused primarily on the conditions for inclusive design and inclusive practices for the initial design phase of regenerative valve implants. While we took regenerative valve implants as a showcase, the norms and design requirements that we identified are (at least in part) more broadly applicable and might therefore also provide guidance for inclusive design of other RM interventions. Additionally, the norms and design requirements we identified are not final and finished but are subject to change, especially given that developments in the RM field are rapidly evolving.

### 4.1. Justice & inclusion: more than implant design

We recognize that regenerative implants do not exist in isolation but are embedded and deployed within wider health system infrastructures. In other words, other factors outside the implant design will also be important to achieve justice and inclusive practices with regards to regenerative implants. For example, health regulation and governance arrangements will have a great influence on the eventual distribution and possibilities for access of certain groups [63]. Also in relation to countering bias in the design of medical devices, systemic changes will be needed in addition to changes to the devices itself, as others have also argued [12,64]. Inclusive design will therefore need to go hand in hand with structural changes at the local (e.g., national governments) and global level (e.g., international organizations and agreements) to promote justice and inclusion in the care available for and received by people around the world.

Particularly when the implant development moves to the next phase of preclinical and clinical testing and implementation in the clinic, the value of justice will raise additional conditions for inclusive practices throughout the cycle of research and care. For example, within *in vivo* and *in vitro* testing data, samples and models that are representative of the user population should be used. Likewise, a representative group of participants should be included in clinical trials. These phases will be crucial for determining which biological and social differences between users are significant and which are not. Further research is necessary to elucidate which additional norms and requirements for inclusive practices are important in the next phases of the development and implementation of regenerative valve implants.

### 4.2. Toward value-sensitive design of regenerative valve implants

While regenerative implants are still under development, researchers, engineers and clinicians can make necessary adjustments and tailor designs to align with various values. We focused particularly on the value of justice for inclusive design of regenerative implants. However, justice is not the sole value of significance, and other values will have to be taken into account. These will introduce additional design requirements.

Other relevant values could for instance include health, safety and autonomy. For each of these values, different conceptualizations are possible. To illustrate, health could be conceptualized as absence of objective pathology, or absence of subjective complaints [65], and autonomy could be understood as individual autonomy or relational autonomy [66]. A promising endeavor for future research would be to extend the approach in this paper to address various other relevant values in the context of regenerative valve implant design.

To make sure that the values and design requirements are supported by those who the design concerns, multiple stakeholders, such as engineers, researchers, clinicians and implant users, should be involved in the process. They could play a role in deliberating which values are relevant for the design of regenerative valve implants, which conceptualization of each value is most appropriate, and how the values should be specified into design requirements. Such deliberation could be a valuable addition to another type of stakeholder involvement, aimed at improving the clinical value of technology, as is being argued for by others in the RM field [67].

An integrated method to systematically address values in the design of a new technology is known as value-sensitive design (VSD) [68,69]. This approach discerns three phases: conceptual investigations, empirical investigations and theoretical investigations, which can be carried out in an iterative and integrative manner [68,69]. The theoretical analysis of the value of justice and its specification into norms and design requirements, as laid out in this paper, corresponds roughly with the conceptual investigations phase. Next steps toward a VSD of regenerative valve implants could then involve empirical investigations to include stakeholder perspectives, conceptual investigations of additional values and values hierarchies, and technical investigations to develop a design that aligns with the identified values, norms and design requirements.

### 4.3. Identifying inter- & intra-value conflicts

Within the pursuit of a VSD for regenerative valve implants, the values may present what appear to be conflicting demands. In other words, they may produce value conflicts [28].

Van de Poel has discerned two types of value conflicts. First, conflicts may arise between values that pose contradictory demands [28]. For example, an inter-value conflict could arise between the values of justice, health and safety in relation to the stratification of the implant design. The values of justice and health may advocate for tailoring the implant design to specific user groups, as proposed under ‘design for difference’. In contrast, safety considerations may recommend the pursuit of a single implant design to streamline safety testing during phase I clinical trials. Second, if multiple stakeholders are involved, the possibility exists that different stakeholders may hold divergent views on which values are relevant for the implant design, or how the values should be conceptualized or specified [28]. In relation to the value of justice, other conceptualizations than the one we described in this paper are possible. According to Van de Poel's description of the activity of conceptualizing values, the choice for one conceptualization of a value over another should be based on two types of considerations: philosophical arguments regarding the adequacy of certain conceptualizations, and practical arguments regarding whether the conceptualization captures the types of impact that the technology design in question may have [28]. We chose a conceptualization of justice that substantiates the aims of inclusive design, which we have argued is relevant for the design regenerative valve implants. Still, stakeholders might disagree on which conceptualization is most appropriate, which would in turn have consequences for the resulting values hierarchy for inclusive design of the implants.

In addition to these two types of value conflicts, a third value conflict was encountered in this paper. Even prior to stakeholder involvement, specification of a value into norms and design requirements might result in design requirements that pose (seemingly) contradictory demands. These could be called intra-value conflicts. For example, a potential conflict may arise between the requirement to ‘design for difference’ and the requirement for ‘affordability and cost–effectiveness’, as stratification – i.e., designing different implants for different user groups – might raise the prices rather than lower them. Choosing between a uniform off-the-shelf available design and a customized design in light of this conflict is a major concern for engineers [37]. Resolving these intra-value conflicts is important because neglecting them can lead to the final implant design harbouring potential injustices, inequalities and inequities. Strategies for resolving value conflicts have been discussed elsewhere [28], and should be taken up in the pursuit of a VSD for regenerative valve implants.

## 5. Conclusion

In this paper, we investigated the conditions for inclusive design of RM interventions, taking regenerative implants as a showcase. We showed that the aim of inclusive design relates to the value of justice. Justice is concerned with recognition (claims about equal respect for marginalized groups and undervalued social identities) and redistribution (the equitable distribution of goods and resources). By means of a value hierarchy, we translated the value of justice into three norms: regenerative valve implants should be designed to promote equal opportunity to good health for all potential users; in the design process, equal respect for all potential users should be shown; and the implants should be designed to be accessible to everyone in need. Based on these norms, we formulated six design requirements: regenerative valve implants should be designed to account for relevant user differences, be affordable and cost-effective and be suitable for global distribution, and the design process should involve diverse design teams, engage users and use inclusive communication.

Overall, by laying down the conditions for inclusive design of regenerative valve implants, we hope to aid the design of implants that are appropriate, respectful and available for everyone in need. More generally, by exemplifying an ethically proactive approach to the design of RM technology, we hope to contribute to responsible development of RM interventions.
